# Association between obstructive sleep apnea and hearing loss among a cohort of emergency responders

**DOI:** 10.1007/s11325-025-03338-8

**Published:** 2025-05-06

**Authors:** David W. Appel, David G. Goldfarb, Rachel Zeig-Owens, Jaeun Choi, Gregory Flamme, Yang Liu, Theresa Schwartz, David J. Prezant

**Affiliations:** 1 The Bureau of Health Services and the FDNY World Trade Center Health Program, Fire Department of the City of New York, Brooklyn, NY USA; 2 Pulmonary Medicine Division, Department of Medicine, Montefiore Medical Center and Albert, Einstein College of Medicine, Bronx, NY USA; 3 Division of Epidemiology, Department of Epidemiology and Population Health, Albert Einstein College of Medicine, Bronx, NY USA; 4 Division of Biostatistics, Department of Epidemiology and Population Health, Albert Einstein College of Medicine, Bronx, NY USA; 5 Stephenson and Stephenson, Research and Consulting, LLC Batavia, Batavia, OH USA

**Keywords:** Berlin questionnaire, Obstructive sleep apnea, Polysomnogram, Audiometry

## Abstract

**Purpose:**

We sought to determine whether risk for obstructive sleep apnea (OSA) and OSA severity are associated with sensorineural hearing loss (HL) among emergency responders.

**Methods:**

We evaluated two independent variables: OSA risk, categorized using Berlin Questionnaire criteria, and OSA severity, determined by polysomnogram (PSG) apnea-hypopnea indices (AHI). Logistic regression, adjusted for confounders, was used to assess the association between each OSA exposure and the outcome of HL among a cohort of emergency responders.

**Results:**

The study cohort included 13,909 participants with audiometric data, 12,834 with Berlin Questionnaire data, and 4,024 participants with PSG data. Those with high and very high OSA risk showed significantly elevated odds of HL at speech frequencies, with adjusted odds ratios (OR) of 1.34 (95% CI: 1.14–1.58; *p* < 0.01) and 1.56 (95% CI: 1.30–1.88; *p* < 0.01), respectively, compared to those with no OSA risk. Combining very high and high risk validated category groupings for the Berlin, those individuals had 41% higher odds for HL over speech frequencies compared to those with no risk (OR = 1.41; 95% CI = 1.21–1.65; *p* < 0.01). Those with PSG-determined severe OSA had higher adjusted odds of HL at speech frequencies than those with no OSA; OR of 1.33 (95% CI: 1.00-1.78; *p* = 0.04).

**Conclusions:**

We report a significant association between OSA and HL among emergency responders. Our results underscore a need for an analysis of the longitudinal association between OSA and HL to identify potential causality and for integrated health interventions that target both conditions in this responder population.

**Supplementary Information:**

The online version contains supplementary material available at 10.1007/s11325-025-03338-8.

## Introduction

Plausible pathophysiological mechanisms linking obstructive sleep apnea (OSA) and sensorineural hearing loss (HL) include: direct effects on the inner ear from hypoxemia that develops during sleep-disordered breathing, injury to the vasculature supplying the inner ear caused by increased redox activity-associated release of inflammatory mediators [1–3] resulting from because of repetitive upper airway closing and reopening, reduced blood flow to the inner ear resulting from frequent sleep arousal-associated noradrenaline release with subsequent vasoconstriction, and acoustic nerve injury resulting from direct effects of snoring related vibration [4–6]. A higher incidence of OSA was reported among patients experiencing sudden HL. [7] In a cross-sectional study of 224 hospital volunteers, researchers found an association between OSA and impaired central auditory function, particularly among older individuals [8]. They hypothesized some of the pathophysiologic mechanisms cited above, but were unable to provide objective supportive data.

A recent meta-analysis of 20 studies (*n* = 34,442 participants) found an association between OSA and HL [9]. Its authors cited weaknesses, however, including several studies with small sample sizes and one large cross-sectional study with numerous unmeasured confounding variables [9]. While their meta-analysis did include a large cross-sectional study (*n* = 13,967) of the Hispanic Community Health Study/Study of Latinos (SOL Study) that controlled for confounders and found an association between OSA and HL, the authors nonetheless noted a need for further research [10.

Our study goals were: (1) to evaluate the association between OSA risk using the Berlin Questionnaire and HL and (2) to evaluate the association between OSA severity determined by polysomnogram (PSG) apnea-hypopnea indices (AHI) and HL.

## Methods and materials

### Study population

**Fig. 1:**
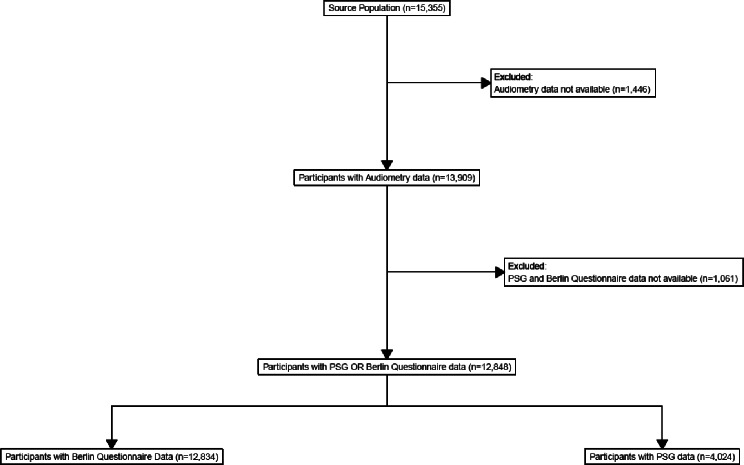
Flow Diagram of Study Participants. Legend: *N* = 14 participants had PSG data but no Berlin Questionnaire data. Abbreviations: PSG = polysomnography

The source population included Fire Department of the City of New York (FDNY) firefighters and Emergency Medical Service (EMS) personnel who responded to the World Trade Center (WTC) attacks. A majority of the cohort provided audiometric and Berlin Questionnaire data, while a sub-sample also performed polysomnography (PSG) (Fig. 1). The Institutional Review Board of the Albert Einstein College of Medicine/Montefiore Medical Center (IRB #07-09-320) approved this study, and all participants provided written informed consent.

### Berlin questionnaire data

Initiated by the FDNY in 2009 as part of participants’ annual medical monitoring, the Berlin Questionnaire, hereafter the “Berlin”, is an instrument that assesses three categories: snoring behavior (category 1), daytime sleepiness (category 2), and the presence of hypertension or obesity (category 3). To receive a high-risk for OSA classification, individuals must provide positive answers in at least two categories. [11] In this study, we classified participants with 0 out of 3, 1 out of 3, 2 out of 3, and 3 out of 3 categories, respectively, as no, low, high, and very high risk for OSA. Our modification departs from the original scoring to allow finer stratification of OSA risk. When participants performed multiple Berlin examinations, we used the Berlin closest in time to the audiometry exam.

### Polysomnography (PSG) data

Shortly after 2009, National Institute of Occupational Safety and Health (NIOSH) authorized providers to order PSG for members suspected to have OSA who also had a recognized WTC associated illness such as asthma or rhinosinusitis [12]. Because the Berlin identifies risk but does not diagnose OSA, we used the subset of participants with PSG testing to confirm the Berlin analyses. Using NIOSH mandated guidelines, FDNY healthcare providers ordered these PSG according to their clinical concerns, usually snoring, choking arousals, witnessed apnea, and excessive daytime sleepiness. Members performed sleep studies between 2003 and 2024 with a majority (66%) performed after 2013. Most participants performed in-laboratory diagnostic or split-night PSG that consisted of standard measures of electroencephalography (EEG), electromyography (EMG), respiratory effort, and pulse oximetry to assess sleep stages, respiratory events (apneas and hypopneas), and oxyhemoglobin saturation. All studies were conducted in American Academy of Sleep Medicine (AASM) accredited laboratories, mostly university affiliated. We classified participants as no OSA (AHI < 5 events/hour), mild OSA (AHI 5–14 events/hour), moderate OSA (AHI 15–29 events/hour) and severe OSA (AHI ≥ 30 events/hour) utilizing AASM Rule 1B scoring criteria [13]. We omitted use of in-lab titration PSG to maintain focus on diagnostic severity evaluation at the initial assessment of OSA. For participants who performed multiple PSG tests, we selected the first diagnostic PSG to capture diagnostic data that most likely reflected their most severe and untreated OSA. A primary author of this paper (DA) reviewed all sleep study reports for data reliability. This review included verifying test type, scoring methodology, and completeness of key variables (e.g., AHI). When data were missing, the reviewer contacted the originating sleep laboratories for clarification. After quality assurance, DA extracted sleep study parameters into a structured database used for all analyses of objective OSA severity.

### Audiometry data

The FDNY Bureau of Health Services conducts medical monitoring examinations for its workforce every 12 to 18 months which includes audiometry examinations. Test environments met ANSI S3.1 with audiometers calibrated according to ANSI S3.6. These examinations included pure-threshold audiograms administered by trained technicians using audiometers calibrated to report thresholds in hearing threshold level through supra-aural earphones within sound booths [14]. We measured thresholds at frequencies of 0.5, 1, 2, 3, 4, 6, and 8 kHz (kHz) in 5-decibel (dB) increments using a modified Hughson-Westlake technique and data underwent quality assurance review, flagging individual thresholds. This is described in greater detail elsewhere [15]. We applied the HL definitions from the Global Burden of Diseases, Injuries, and Risk Factors (GBD) Study [16]. HL was defined separately as speech frequency loss and as high frequency loss. Speech frequency HL was computed by averaging the lowest audible sound in the better ear across frequencies of 0.5, 1, 2, and 4 kHz, as these frequencies cover most speech ranges and provide a standardized measure. Specifically, we calculated averages in these frequencies for each ear and used the lower average for analysis. HL for speech frequencies was defined as an average hearing threshold greater than 20-dB Hearing Threshold Level (HTL). Separately, we computed high frequency HL, using the average thresholds at 3, 4, and 6 kHz in the better ear. Given that high-frequency HL tends to occur at a younger age, HL for high frequencies was defined as an average threshold greater than 35-dB HTL. For participants with multiple audiometry exams during the period between 9/11/2001 and 12/31/2017, we selected the most recent exam to use in analyses.

### Demographic and covariate data

We obtained demographic information such as birth date, race/ethnicity, and sex from the FDNY employee database. During FDNY routine annual medical monitoring exams we measured height and weight and collected self-reported smoking status (ever or never). We calculated body mass index (BMI) and categorized participants as underweight/normal (< 25 kg/m²), overweight (25–29.9 kg/m²), and obese (≥ 30 kg/m²).

### Statistical analyses

As appropriate, we summarized descriptive characteristics for the study cohort using means, standard deviations, counts, and proportions (Table 1). We reported additional descriptive statistics for participants who underwent sleep studies with PSG testing.

**Table 1:** Selected characteristics of study cohort

	Overall (*N* = 12,848)	Berlin only (*N* = 8,824)	PSG* (*N* = 4,024)
**Age at hearing exam**			
Mean (SD)	49.9 (7.8)	50.3 (8.1)	48.9 (7.0)
**Race**			
White	11,269 (87.7%)	7,590 (86.0%)	3,679 (91.4%)
Black	642 (5.0%)	515 (5.8%)	127 (3.2%)
Hispanic	833 (6.5%)	632 (7.2%)	201 (5.0%)
Other	104 (0.8%)	87 (1.0%)	17 (0.4%)
**Sex**			
Male	12,476 (97.1%)	8,525 (96.6%)	3,951 (98.2%)
Female	372 (2.9%)	299 (3.4%)	73 (1.8%)
**BMI Category**			
Underweight: <18.5 kg/m²	3 (0.0%)	3 (0.0%)	0 (0.0%)
Normal: 18.5–24.9 kg/m²	1062 (8.3%)	876 (9.9%)	189 (4.7%)
Overweight: 25.0–29.9 kg/m²	5,698 (44.4%)	4,244 (48.1%)	1,453 (36.1%)
Obese: ≥30.0 kg/m²	6,085 (47.4%)	3,704 (42.0%)	2,382 (59.2%)
**Work assignment**			
Firefighter	11,200 (87.2%)^§^	7,472 (84.7%)^§^	3,728 (92.6%)
EMS	1,647 (12.8%)	1,351 (15.3%)	296 (7.4%)
**Ever smoker**			
Never	8,315 (64.7%)	5,602 (63.4%)	2,716 (67.5%)
Current or Former	4,533 (35.3%)	3,230 (36.6%)	1,308 (32.5%)
**Years of service**			
Mean (SD)	22.8 (7.3)	22.9 (7.4)	22.6 (7.1)
**WTC Arrival Time**			
Morning of 9/11/2001	2,029 (15.8%)	1,338 (15.2%)	691 (17.2%)
Afternoon of 9/11/2001	6,128 (47.7%)	4,073 (46.2%)	2,055 (51.1%)
9/12/2001	2,296 (17.9%)	1,573 (17.8%)	723 (18.0%)
9/13/2001-9/24/2001	2,069 (16.1%)	1,566 (17.8%)	503 (12.5%)
After 9/24/2001	326 (2.5%)	274 (3.1%)	52 (1.3%)
**Berlin OSA Risk (# categories)**			
None (0/3)	2002 (15.6%)	1,886 (21.4%)	117 (2.9%)
Low Risk (1/3)	3818 (29.7%)	3,161 (35.8%)	655 (16.3%)
High Risk (2/3)	4430 (34.5%)	2,725 (30.9%)	1,709 (42.6%)
Very High Risk (3/3)	2583 (20.1%)	1,052 (11.9%)	1,529 (38.1%)
**AHI-based OSA severity**			
None (AHI: 0–4)	837 (20.8%)	n/a	837 (20.8%)
Mild (AHI: 5–14)	1,132 (28.1%)	n/a	1,132 (28.1%)
Moderate (AHI: 15–29)	914 (22.7%)	n/a	914 (22.7%)
Severe (AHI: ≥30)	1,141 (28.4%)	n/a	1141 (28.4%)
**≥ 20 dB 0.5**,**1**,**2**,**4 kHz average**			
No	10,974 (85.4%)	7,499 (85.0%)	3,475 (86.4%)
Yes	1,874 (14.6%)	1,325 (15.0%)	549 (13.6%)
**≥ 35 dB 3**,**4**,**6 kHz average**			
No	10,761 (83.8%)	7,326 (83.0%)	3,435 (85.4%)
Yes	2,087 (16.2%)	1,498 (17.0%)	589 (14.6%)

Abbreviations: Berlin = Berlin Questionnaire; PSG = polysomnography; AHI = Apnea Hypopnea Index; EMS = Emergency Medical Service Provider; kHz = kilohertz; kg = kilograms; m = meters; BMI = body mass index; dB = decibels; SD = standard deviation

AHI is expressed as events per hour

*n = 14 participants had a PSG only but no Berlin Questionnaire

‡Denominator for AHI category in overall column is 4,024

^§^*n* = 1 civilian

First, using logistic regression we examined the association of OSA risk indicated by the Berlin and HL among all participants, including those who did not perform a PSG. We used Berlin OSA risk status (none, low, high, very high, defined above) in the models as a categorical variable with “none” as the reference category. We used HL at speech frequencies as the outcome for the primary analyses and HL at high frequencies for secondary analyses. We adjusted models for potential confounders selected on theory and past literature: white vs. non-white, age at hearing examination, sex, years of service, cigarette smoking, and WTC arrival time for exposure assessment effect. We conducted two secondary analyses: (1) we adjusted the model for BMI as an additional potential confounder and removed WTC arrival time from the covariate set, and (2) we examined the association between OSA risk categorized according to the validated Berlin Questionnaire classification. Specifically, in this analysis we classified participants with 0 out of 3, 1 out of 3, and 2 or 3 out of 3 categories, respectively, as no, low, and high/very high risk for OSA.

Using the same HL outcomes among our subset of participants who performed PSG, we conducted logistic regression to examine OSA severity derived by PSG and HL relationships, adjusting for BMI along with the abovementioned confounders. In a secondary analysis, we used a dichotomous variable to indicate the presence or absence of OSA, defining OSA as an AHI of 5 or more events per hour (i.e., mild or worse) versus no OSA. To assess the stability of Berlin-risk classification over time, we calculated the proportion of instances where a patient’s risk status remained consistent in more than 50% of their repeated measurements.

## Results

The source population included 15,355 firefighters and EMS employed by FDNY, all of whom responded to the WTC attacks. We obtained audiometric information from 13,909 (91.3%) of the source population. Among those with at least one audiometry exam, 12,834 (92.4%) participants completed a Berlin, and 4,024 (28.7%) performed PSG (Fig. 1). Table 1 presents descriptive characteristics for the study cohort for participants with a Berlin, a PSG, and overall. 8,824 participants had only a Berlin, 4,010 had both a Berlin and a PSG. The mean age at the hearing exam was 50.3 years for the Berlin-only group and 48.9 years for the PSG group, with an overall mean of 49.9 years. Most participants were white (87.7%) and male (97.1%), with firefighters (87.2%) comprising 84.7% of the Berlin-only group and 92.7% of the PSG group. HL, defined as ≥ 20-dB at 0.5, 1, 2, 4 kHz, occurred in 1,874 study participants (14.6%) and HL, defined as ≥ 35-dB at 3, 4, and 6 kHz, occurred in 16.2% of the overall population, with slightly higher rates in the Berlin-only group (17.0%) compared to the PSG group (14.7%). Descriptive characteristics by AHI-based OSA severity can be found in Supplemental Table S1. Among those with PSG-confirmed severe OSA (AHI ≥ 30; *n* = 1,141), the Berlin categorized 90% at risk for OSA − 51% and 39% with very high risk and high risk for OSA, respectively. The prevalence of HL (≥ 20-dB HTL) at lower-frequency speech thresholds (0.5, 1, 2, 4 kHz average) increased with PSG-derived OSA severity: 11%, 13%, 14%, and 17% for none, mild, moderate, and severe OSA, respectively. We observed a similar pattern for high-frequency HL (3, 4, 6 kHz). Of all participants who completed the Berlin questionnaire, 7,800 (60.8%) scored in the first domain related to snoring, 4,611 (35.9%) scored in the second domain concerning fatigue, and 7,207 (56.2%) scored in the third domain indicating self-reported high blood pressure or obesity. The Berlin-risk classification remained stable in 76.0% of patients; i.e., they retained the same status in the majority of their measurements.

### Berlin questionnaire identified OSA risk and HL

**Fig. 2:**
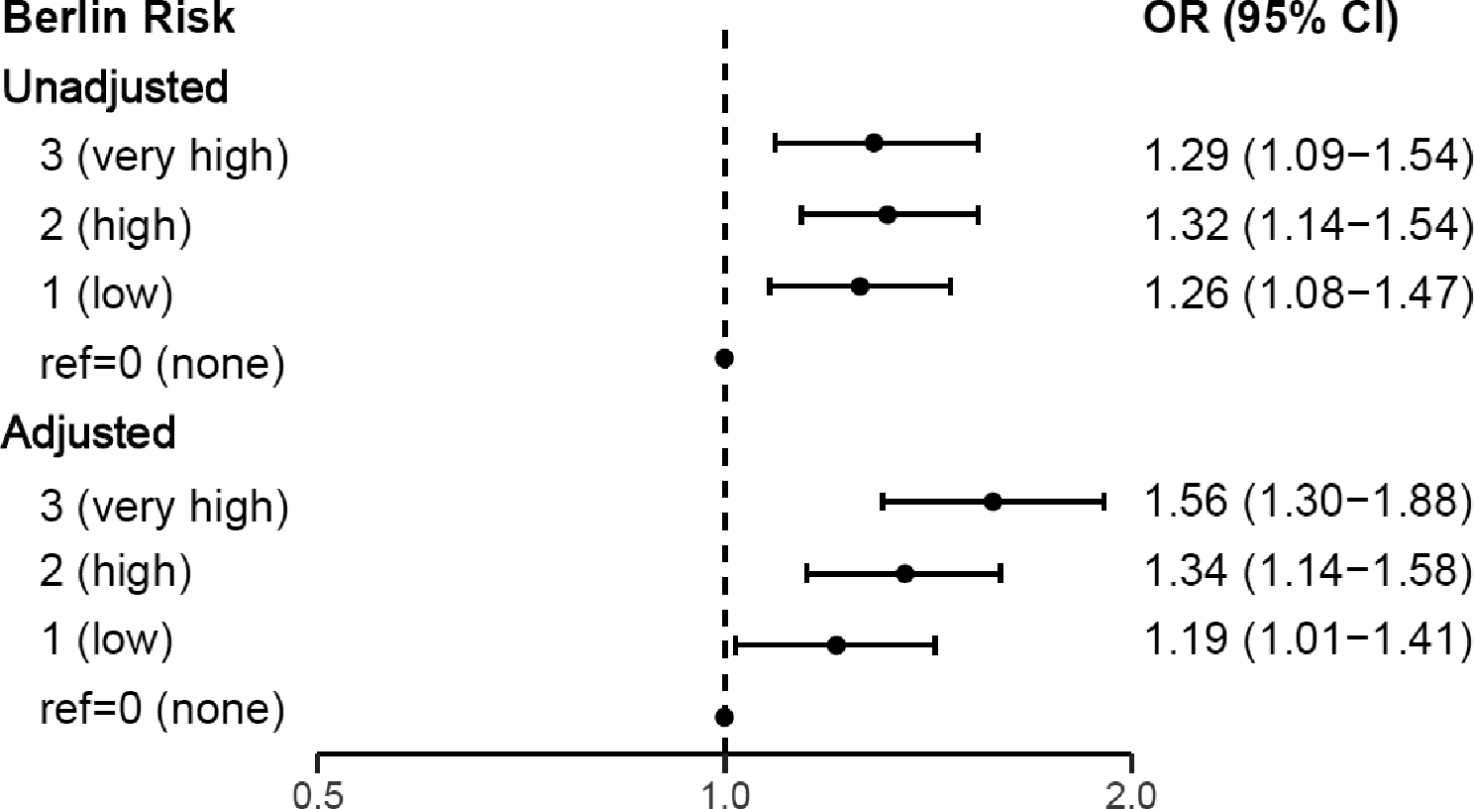
Evaluating the association between Berlin Questionnaire OSA Risk and Hearing Loss at Speech Frequencies. Abbreviations: kHz = kilohertz^;^ The adjusted models account for age at the time of the audiometry exam, race (white vs. non-white), sex, years of service, arrival time at the WTC site, and smoking status (ever vs. never). Speech frequency HL was computed by averaging the lowest audible sound in the better ear across frequencies of 0.5 kHz, 1 kHz, 2 kHz, and 4 kHz. Outcome measures included hearing loss (≥ 20 decibels (dB)) at speech frequency averages

We used logistic regression models to explore the link between OSA risk (Berlin), and HL across all participants (Fig. 2, S2 and S4). After adjusting for confounders, individuals scoring high (2/3 categories) and very high risk (3/3 categories) on the Berlin had 34% (OR = 1.34; 95% CI = 1.14–1.58; *p* < 0.01) and 56% (OR = 1.56; 95% CI = 1.30–1.88; *p* < 0.01) higher odds for HL (≥ 20-dB HTL) over speech frequencies (0.5–4 Hz) compared to those at no risk. In our secondary analysis that adjusted for BMI and excluded WTC arrival time as a covariate, we observed increased odds of HL at speech frequencies for individuals classified as high risk (OR = 1.21; 95% CI: 1.02–1.45; *p* = 0.03) and very high risk (OR = 1.36; 95% CI: 1.10–1.67; *p* < 0.01), respectively. Combining very high and high risk validated category groupings for the Berlin, those individuals had 41% higher odds for HL (≥ 20-dB HTL) over speech frequencies (0.5–4 Hz) compared to those with no risk (OR = 1.41; 95% CI = 1.21–1.65; *p* < 0.01).

Similarly, those with high and very high risk on the Berlin showed 14% (OR = 1.14; 95% CI = 0.98–1.34; *p* = 0.09) and 35% (OR = 1.35; 95% CI = 1.13–1.61; *p* < 0.01) greater adjusted odds for high-frequency HL (≥ 35-dB HTL) than those at no risk (Figure S2).

### PSG identified OSA and HL

**Fig. 3:**
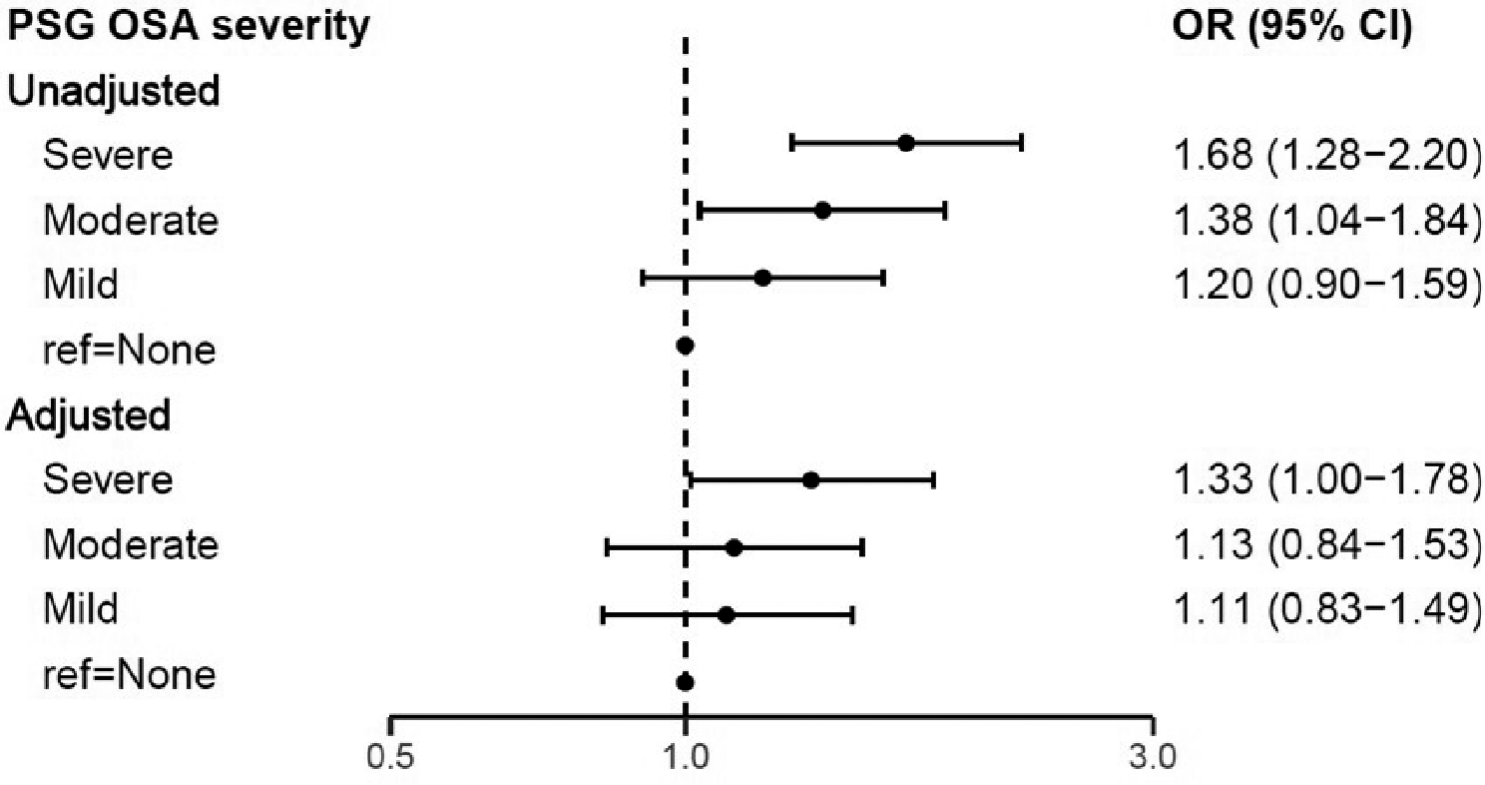
Evaluating the association between OSA severity by PSG and Hearing Loss at Speech Frequencies. Abbreviations: OSA = Obstructive Sleep Apnea, PSG = Polysomnography, kHz = kilohertz. The Apnea-Hypopnea Index (AHI) was subsequently calculated as the average number of apneas and hypopneas per hour of sleep, classifying OSA severity as no OSA (AHI < 5), mild (AHI 5–14), moderate (AHI 15–29), and severe (AHI ≥ 30). The adjusted models account for BMI, age at the time of the PSG, race (white vs. non-white), sex, years of service, arrival time at the WTC site and smoking status (ever vs. never). Speech frequency HL was computed by averaging the lowest audible sound in the better ear across frequencies of 0.5 kHz, 1 kHz, 2 kHz, and 4 kHz. Outcome measures included hearing loss (≥ 20 decibels (dB)) at speech frequency averages

Logistic regression assessed the relationship between OSA severity measured by PSG and HL among participants who performed a PSG (Fig. 3, S3, and S5). Those with PSG-derived severe OSA had 33% (adjusted OR = 1.33; 95% Cl 1.00-1.78; *p* = 0.04) higher odds for HL over speech frequencies (0.5–4 kHz) compared to those with no OSA. We observed 48% and 37% increased unadjusted odds of high frequency HL for those with severe and moderate OSA, respectively. Adjusting for confounders, notably BMI, attenuated this association, however, the trend showing increasing odds of HL for more severe OSA persisted. In our secondary analysis using the dichotomous definition, the presence of any OSA (AHI ≥ 5) associated with greater odds of HL at speech frequencies (adjusted OR = 1.26; 95% Cl 0.99–1.66; *p* = 0.06).

## Discussion

In this cross-sectional study of 12,848 FDNY emergency responders, we show a consistent pattern: in dose-response trends, as the risk and actual severity of OSA increased, so rose the likelihood of HL. We found that individuals with high and very high OSA risk had 34% and 56% higher adjusted odds, respectively, of mild to profound hearing loss across both speech frequencies (0.5–4 kHz) and high frequencies (3–6 kHz) than those at no risk (Objective 1). Among the cohort subset who performed PSG sleep studies, those with severe OSA (AHI ≥ 30 events/hour) had 33% increased adjusted odds for HL at speech frequencies than those with no OSA (Objective 2). We found a similar association between moderately severe OSA (AHI = 15–29 events/hour) and HL, however, adjusting for confounders attenuated this association. While we modified the Berlin risk categories from 3 to 4 (i.e., separating high and very high risk) to identify those with especially high risk, retaining the validated risk categories did not alter the association between Berlin risk for OSA and HL.

We studied a mostly male, white, overweight/obese cohort substantially younger than those recently described in a New England Journal of Medicine review article, but similar in age to those in many studies that reported OSA-HL associations [17]. We adjusted for many variables that affect hearing and OSA including age and BMI. Earlier studies of much smaller cohorts of responders followed over shorter periods showed associations between WTC arrival time, a proxy for exposure, and both HL and OSA. In our current study of a substantially larger cohort monitored over longer periods, we sought an OSA-HL association independent of WTC exposure by controlling for WTC arrival time. Our adjusted analyses, the demographic differences between ours and the HCHS/SOL cohorts, [10] and the resonance of findings across these two large studies justify our claim for a true OSA-HL association that is generalizable to the population at large.

OSA associated with increased likelihood of HL across a range of frequencies. We note the strongest association for HL in the low to mid frequency ranges (often referred to as the speech range). Importantly, we noted increased mean hearing thresholds at each sound frequency of interest (low and high frequencies) among those with severe OSA and a similar tendency among those with moderate OSA, but not with mild or no OSA.

The Berlin is validated to identify those with high or low risk of OSA. We found that high risk for OSA (classical non-modified Berlin) and high risk/very high risk (modified Berlin) associated strongly with HL, while no risk for OSA did not. Our high/very high-HL associations derive from careful data collection and analysis from a large cohort, however, the Berlin was not designed to evaluate outcomes beyond OSA risk. Therefore, our observation of an OSA-HL association warrants cautious interpretation and further investigation. We found no association for no risk and moderate risk for OSA and HL (modified Berlin). The Berlin moderate risk for OSA trended toward an association with HL, though the effect did not reach statistical significance. To our knowledge, this study is the first to identify an association between Berlin Questionnaire scores and HL. We modified the Berlin, thinking that doing so could produce greater precision with regard to an association with HL. While the Berlin was not designed to evaluate outcomes beyond OSA risk, we do believe that we found highly sensitive associations of Berlin risk assessment with both PSG findings and HL. Thus, we believe this supports careful use of Berlin as a proxy for PSG-AHI in future longitudinal studies. Such studies will require diligent data collection and careful cautious interpretation.

Potential pathophysiologic mechanisms, referenced in our introduction, contribute to hypoxic injury of the inner ear and may be influenced by elevated BMI, which is associated with both OSA and HL. The current study did not evaluate the likelihoods of specific causal mechanisms. However, our Berlin data support our belief that OSA developed among our participants years before their PSG diagnosis. Two small studies examining continuous positive airway pressure (CPAP) treatment failed to find a favorable impact on HL [18, 19]. Notably, however, if OSA contributes to degeneration of inner ear hair cells, such damage is not reversible in humans, underscoring prevention rather than repair as the primary therapeutic objective. Early identification of individuals at heightened risk for both OSA and HL—through screening tools such as the Berlin questionnaire—may facilitate timely intervention to prevent irreversible cochlear hair cell damage.

Our study has limitations. First, its cross-sectional design precludes making causal claims regarding the association between OSA risk or confirmed OSA and HL. It should be noted, however, that our study possesses many of the criteria used for assessing causality [20, 21] including strength (effect size), consistency with other studies, dose-response relationships and bio-plausibility. The entirety of these criteria applies to our study. Second, our inability to account for occupational noise exposure and ambient noise exposure at audiometry exams could confound the observed association between OSA and HL. Third, while we found an association between severe OSA and HL, no significant association was observed for mild or moderate OSA. This could reflect limited statistical power or a true lack of effect at lower severity levels, but it does limit the generalizability of our findings across the full OSA severity spectrum. Fourth, while we provide no data regarding pathophysiologic mechanisms that associate OSA with HL, our Berlin data support our belief that OSA developed in our participants years before their PSG diagnosis (and years before HL). Use of the Berlin as a proxy for PSG-AHI in our longitudinal studies may identify sooner those with severe OSA to facilitate OSA treatment and modify HL. Lastly, our study lacks longitudinal outcome analyses, an area we plan for future investigation to provide further insights into the relationship over time and the impact OSA treatment could have on HL.

This study has several strengths. It is the first of its kind to explore the association between OSA and HL in an occupational/environmental cohort. As such, our data, adjusted for important confounders, add to those from previous reports showing an OSA-HL association. Its strengths include cohort size, high-quality audiometry, “gold-standard” PSG-derived AHI; all which were quality assured. Perhaps more importantly, to our knowledge this study is the first to identify an association between Berlin scores and HL. This has considerable pragmatic value because of the relative ease of determining a Berlin score. Further, members’ Berlin score categories tended to remain the same over years. This raises the possibility for a role, used cautiously, for Berlin risk as a proxy for OSA severity in future longitudinal studies.

To conclude, OSA and HL are highly prevalent and treatable conditions that left untreated may lead to adverse cardiovascular and cognitive outcomes [22–29]. Cross-sectional studies controlled for confounders from FDNY and HCHS/SOL^10^ now demonstrate a dose-response effect of OSA severity on increased HL with over 12,000 participants in the current FDNY study and nearly 14,000 participants in the HCHS/SOL study. Given our findings in two large and very different cohorts, we believe that health evaluation designs should acknowledge and assess this OSA-HL association. Application of the Berlin, with its relative ease of use, may facilitate such an assessment. Longitudinal studies of the Berlin may lead to early identification of those with OSA, HL, and possibly other conditions. We believe it appropriate for clinicians considering OSA in their patients to also inquire about hearing and similarly for their patients with HL to administer the Berlin.

## Electronic supplementary material

Below is the link to the electronic supplementary material.

Supplementary Material 1

## Data Availability

The datasets generated during and/or analyzed during the current study are available from the corresponding author on reasonable request.

## Abbreviations

AHI: Apnea Hypopnea Index
AASM: American Academy of Sleep Medicine
BMI: Body Mass Index
dB: Decibels
EMS: Emergency Medical Service Provider
FDNY: Fire Department of the City of New York
GBD: Global Burden of Diseases, Injuries, and Risk Factors Study
HL: Hearing Loss
HTL: Hearing Threshold Level
kHz: Kilohertz
OSA: Obstructive Sleep Apnea
PSG: Polysomnography
SD: Standard Deviation
SOL Study: Hispanic Community Health Study/Study of Latinos
WTC: World Trade Center

## References

[1] Kheirandish-Gozal L, Gozal D (2019) Obstructive sleep apnea and inflammation: proof of concept based on two illustrative cytokines. 20(3):459 10.3390/ijms20030459 PMC6387387 30678164

[2] Lavie L (2003) Obstructive sleep Apnoea syndrome–an oxidative stress disorder. Sleep Med Rev 7(1):35–51 12586529 10.1053/smrv.2002.0261

[3] Yokoe T, Minoguchi K, Matsuo H et al (2003) Elevated levels of C-reactive protein and interleukin-6 in patients with obstructive sleep apnea syndrome are decreased by nasal continuous positive airway pressure. Circulation 107(8):1129–1134 12615790 10.1161/01.cir.0000052627.99976.18

[4] Kim JB, Seo BS, Kim JH (2019) Effect of arousal on sympathetic overactivity in patients with obstructive sleep apnea. Sleep Med 62:86–91 30975558 10.1016/j.sleep.2019.01.044

[5] Somers VK, Dyken ME, Clary MP, Abboud FM (1995) Sympathetic neural mechanisms in obstructive sleep apnea. J Clin Invest 96(4):1897–1904 7560081 10.1172/JCI118235 PMC185826

[6] Naughton MT, Benard DC, Liu PP, Rutherford R, Rankin F, Bradley TD (1995) Effects of nasal CPAP on sympathetic activity in patients with heart failure and central sleep apnea. Am J Respir Crit Care Med 152(2):473–479 7633695 10.1164/ajrccm.152.2.7633695

[7] Sheu JJ, Wu CS, Lin HC (2012) Association between obstructive sleep apnea and sudden sensorineural hearing loss: a population-based case-control study. Arch Otolaryngol Head Neck Surg 138(1):55–59 22249630 10.1001/archoto.2011.227

[8] Hwang JH, Chen JC, Hsu CJ, Liu TC (2011) Association of obstructive sleep apnea and auditory dysfunctions in older subjects. Otolaryngol Head Neck Surg 144(1):114–119 21493399 10.1177/0194599810390859

[9] Kasemsuk N, Chayopasakul V, Banhiran W et al (2023) Obstructive sleep apnea and sensorineural hearing loss: A systematic review and Meta-analysis. Otolaryngol Head Neck Surg 169(2):201–209 36040818 10.1177/01945998221120777

[10] Chopra A, Jung M, Kaplan RC et al (2016) Sleep apnea is associated with hearing impairment: the Hispanic community health study/study of Latinos. J Clin Sleep Med 12(5):719–726 26951413 10.5664/jcsm.5804 PMC4865559

[11] Netzer NC, Stoohs RA, Netzer CM, Clark K, Strohl KP (1999) Using the Berlin questionnaire to identify patients at risk for the sleep apnea syndrome. Ann Intern Med 131(7):485–491 10507956 10.7326/0003-4819-131-7-199910050-00002

[12] Centers for Disease Control and Prevention World Trade Center Health Program What conditions are covered by the Program? 2022. Located at: https://www.cdc.gov/wtc/pdfs/coveredConditions/WTCHP_CoveredConditions_ENG-508.pdf​.

[13] American Academy of Sleep Medicine AASM releases updated version of scoring manual. https://aasm.org/aasm-releases-updated-version-scoring-manual/

[14] Carhart R, Jerger JF (1959) Preferred method for clinical determination of Pure-Tone thresholds. J Speech Hear Disorders 24(4):330–345

[15] Flamme GA, Goldfarb DG, Zeig-Owens R et al (2019) Hearing loss among world trade center firefighters and emergency medical service workers. J Occup Environ Med 61(12):996–1003 31567659 10.1097/JOM.0000000000001717

[16] GBD 2019 Hearing Loss Collaborators (2021) Hearing loss prevalence and years lived with disability, 1990–2019: findings from the global burden of disease study 2019. Lancet 397(10278):996–1009 33714390 10.1016/S0140-6736(21)00516-X PMC7960691

[17] Lin FR (2024) Age-Related hearing loss. N Engl J Med 390(16):1505–1512 38657246 10.1056/NEJMcp2306778

[18] Chi JC, Lee SD, Huang RJ et al (2021) CPAP treatment improves pure tone audiometry threshold in sensorineural hearing loss patients with Sleep-Disordered breathing. Int J Environ Res Public Health.;18(13) 10.3390/ijerph18136768 PMC8297118 34202447

[19] Nakayama M, Masuda A, Ando KB et al (2015) A pilot study on the efficacy of continuous positive airway pressure on the manifestations of Meniere’s disease in patients with concomitant obstructive sleep apnea syndrome. J Clin Sleep Med 11(10):1101–1107 26094927 10.5664/jcsm.5080 PMC4582051

[20] Hill AB (1965) The environment and disease: association or causation?? Proc R Soc Med 58(5):295–300 14283879 10.1177/003591576505800503 PMC1898525

[21] Shimonovich M, Pearce A, Thomson H, Keyes K, Katikireddi SV (2021) Assessing causality in epidemiology: revisiting Bradford hill to incorporate developments in causal thinking. Eur J Epidemiol 36(9):873–887 33324996 10.1007/s10654-020-00703-7 PMC8206235

[22] Yaggi HK, Concato J, Kernan WN, Lichtman JH, Brass LM, Mohsenin V (2005) Obstructive sleep apnea as a risk factor for stroke and death. N Engl J Med 353(19):2034–2041 16282178 10.1056/NEJMoa043104

[23] Shahar E, Whitney CW, Redline S et al (2001) Sleep-disordered breathing and cardiovascular disease: cross-sectional results of the sleep heart health study. Am J Respir Crit Care Med 163(1):19–25 11208620 10.1164/ajrccm.163.1.2001008

[24] Marin JM, Carrizo SJ, Vicente E, Agusti AG (2005) Long-term cardiovascular outcomes in men with obstructive sleep apnoea-hypopnoea with or without treatment with continuous positive airway pressure: an observational study. Lancet 365(9464):1046–1053 15781100 10.1016/S0140-6736(05)71141-7

[25] Lin FR, Yaffe K, Xia J et al (2013) Hearing loss and cognitive decline in older adults. JAMA Intern Med 173(4):293–299 23337978 10.1001/jamainternmed.2013.1868 PMC3869227

[26] Jean-Louis G, Zizi F, Clark LT, Brown CD, McFarlane SI (2008) Obstructive sleep apnea and cardiovascular disease: role of the metabolic syndrome and its components. J Clin Sleep Med 4(3):261–272 18595441 PMC2546461

[27] Gami AS, Pressman G, Caples SM et al (2004) Association of atrial fibrillation and obstructive sleep apnea. Circulation 110(4):364–367 15249509 10.1161/01.CIR.0000136587.68725.8E

[28] Bisogno A, Scarpa A, Di Girolamo S et al (2021) Hearing loss and cognitive impairment: epidemiology, common pathophysiological findings, and treatment considerations. Life (Basel).;11(10) 10.3390/life11101102 PMC8538578 34685474

[29] Anandam A, Patil M, Akinnusi M, Jaoude P, El-Solh AA (2013) Cardiovascular mortality in obstructive sleep Apnoea treated with continuous positive airway pressure or oral appliance: an observational study. Respirology 18(8):1184–1190 23731062 10.1111/resp.12140

