# Supplementary material for: Association between obstructive sleep apnea and hearing loss among a cohort of emergency responders
Source: Sleep Breath. 2025 May 6;29(2):177. doi: 10.1007/s11325-025-03338-8 (PMC12055870; doi:10.1007/s11325-025-03338-8)
Supplement: Supplementary file 1 — Supplementary Material 1 [file 11325_2025_3338_MOESM1_ESM.docx]

**Supplemental table S1: Descriptive characteristics by AHI-based OSA severity**

|  | **None (AHI:0-4) (N=837)** | **Mild (AHI:5-14) (N=1132)** | **Moderate (AHI:15-29) (N=914)** | **Severe**  **(AHI: ≥30) (N=1141)** |  |
| --- | --- | --- | --- | --- | --- |
| **Age at hearing exam** |  |  |  |  |  |
| Mean (SD) | 48.1 (6.94) | 48.4 (6.74) | 49.2 (7.07) | 49.6 (7.29) |  |
| **Race** |  |  |  |  |  |
| White | 764 (91.3%) | 1042 (92.1%) | 836 (91.5%) | 1037 (90.9%) |  |
| Black | 25 (3.0%) | 33 (2.9%) | 25 (2.7%) | 44 (3.9%) |  |
| Hispanic | 45 (5.4%) | 55 (4.8%) | 45 (4.9%) | 56 (4.9%) |  |
| Other | 3 (0.4%) | 2 (0.2%) | 8 (0.9%) | 4 (0.4%) |  |
| **Sex** |  |  |  |  |  |
| Male | 811 (96.9%) | 1103 (97.4%) | 907 (99.2%) | 11330 (99.0%) |  |
| Female | 26 (3.1%) | 29 (2.6%) | 7 (0.8%) | 11 (1.0%) |  |
| **Work assignment** |  |  |  |  |  |
| Firefighter | 789 (94.3%) | 1051 (92.8%) | 855 (93.5%) | 1033 (90.5%) |  |
| EMS | 48 (5.7%) | 81 (7.2%) | 59 (6.5%) | 108 (9.5%) |  |
| **BMI Category** |  |  |  |  |  |
| Underweight: <18.5 kg/m² | 0 (0%) | 0 (0%) | 0 (0%) | 0 (0%) |  |
| Normal: 18.5-24.9 kg/m² | 69 (8.2%) | 60 (5.3%) | 45 (4.9%) | 15 (1.3%) |  |
| Overweight: 25.0-29.9 kg/m² | 373 (44.6%) | 481 (42.5%) | 321 (35.1%) | 278 (24.4%) |  |
| Obese: ≥30.0 kg/m² | 395 (47.2%) | 591 (52.2%) | 548 (60.0%) | 848 (74.3%) |  |
| **Berlin Risk (# categories)*** |  |  |  |  |  |
| None (0/3) | 43 (5.2%) | 47 (4.2%) | 20 (2.2%) | 7 (0.6%) |  |
| Low Risk (1/3) | 183 (21.9%) | 218 (19.3%) | 144 (15.8%) | 110 (9.7%) |  |
| Moderate Risk (2/3) | 352 (42.2%) | 491 (43.5%) | 422 (46.4%) | 444 (39.1%) |  |
| Very high Risk (3/3) | 257 (30.8%) | 372 (33.0%) | 324 (35.6%) | 576 (50.7%) |  |
| **Ever smoker** |  |  |  |  |  |
| Never | 577 (68.9%) | 797 (70.4%) | 602 (65.9%) | 740 (64.9) |  |
| Current or Former | 260 (31.1%) | 335 (29.6%) | 312 (34.1%) | 401 (35.1%) |  |
| **≥20 dB 0.5,1,2,4 kHz average** |  |  |  |  |  |
| No | 748 (89.4%) | 991 (87.5%) | 785 (85.9%) | 951 (83.4%) |  |
| Yes | 89 (10.6%) | 141 (12.5%) | 129 (14.1%) | 190 (16.7%) |  |
| **≥35 dB 3,4,6 kHz average** |  |  |  |  |  |
| No | 732 (87.5%) | 998 (88.2%) | 764 (83.6%) | 941 (82.5%) |  |
| Yes | 105 (12.5%) | 134 (11.9%) | 150 (16.4%) | 200 (17.5%) |  |
| Abbreviations: Berlin=Berlin Questionnaire; PSG=polysomnography; AHI=Apnea Hypopnea Index; EMS=Emergency Medical Service Provider; kHz=kilohertz; kg=kilograms; m=meters; BMI=body mass index; dB=decibels; SD=standard deviation AHI is expressed as events per hour.  *The Berlin Questionnaire was missing for 2 individuals in the 'none' OSA group, 4 individuals in the mild OSA group, 4 individuals in the moderate OSA group, and 4 individuals in the severe OSA group. | | | | | |

**Supplemental table S2: Evaluating the association between Berlin Questionnaire OSA Risk and High Frequency Hearing Loss**

**
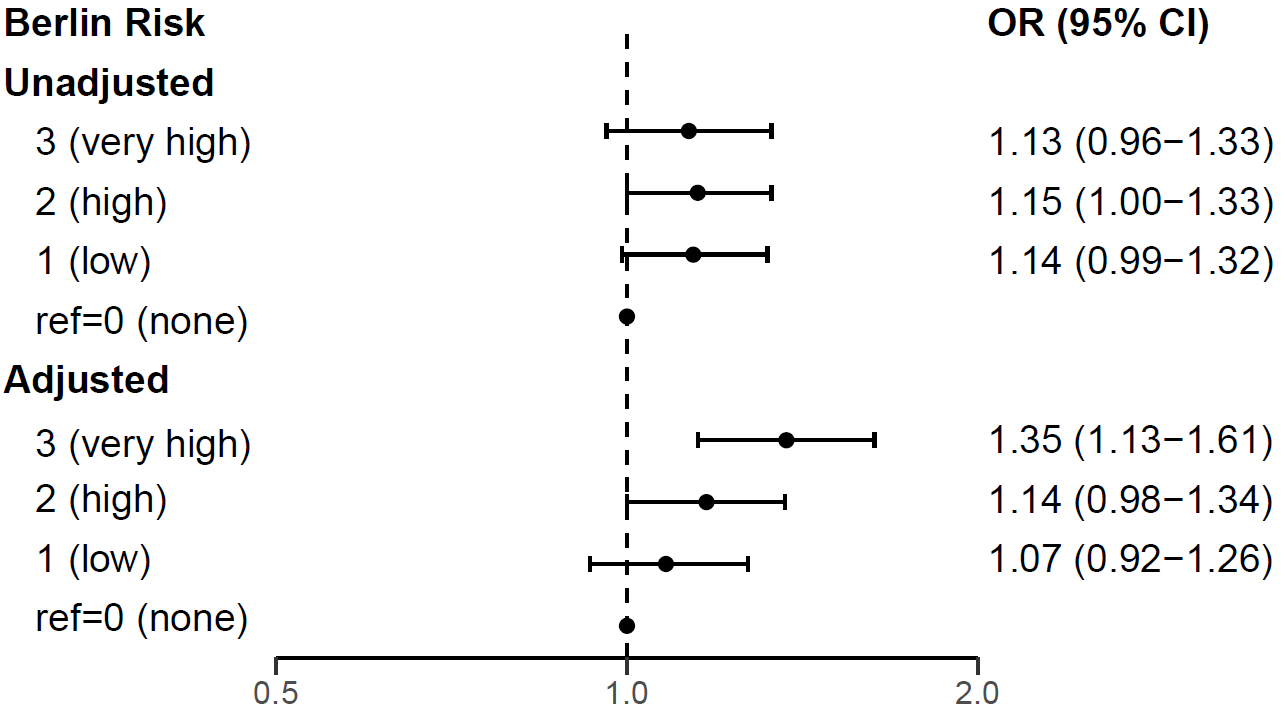
**

The adjusted models account for age at the time of the audiometry exam, race (white vs. non-white), sex, years of service, arrival time at the WTC site, and smoking status (ever vs. never). High frequency HL was computed by averaging the lowest audible sound in the better ear across frequencies of 3 kHz, 4 kHz, and 6 kHz. Outcome measures included high-frequency hearing loss (≥35 dB).

**Supplemental table S3: Evaluating the association between OSA severity by PSG and High Frequency Hearing Loss**


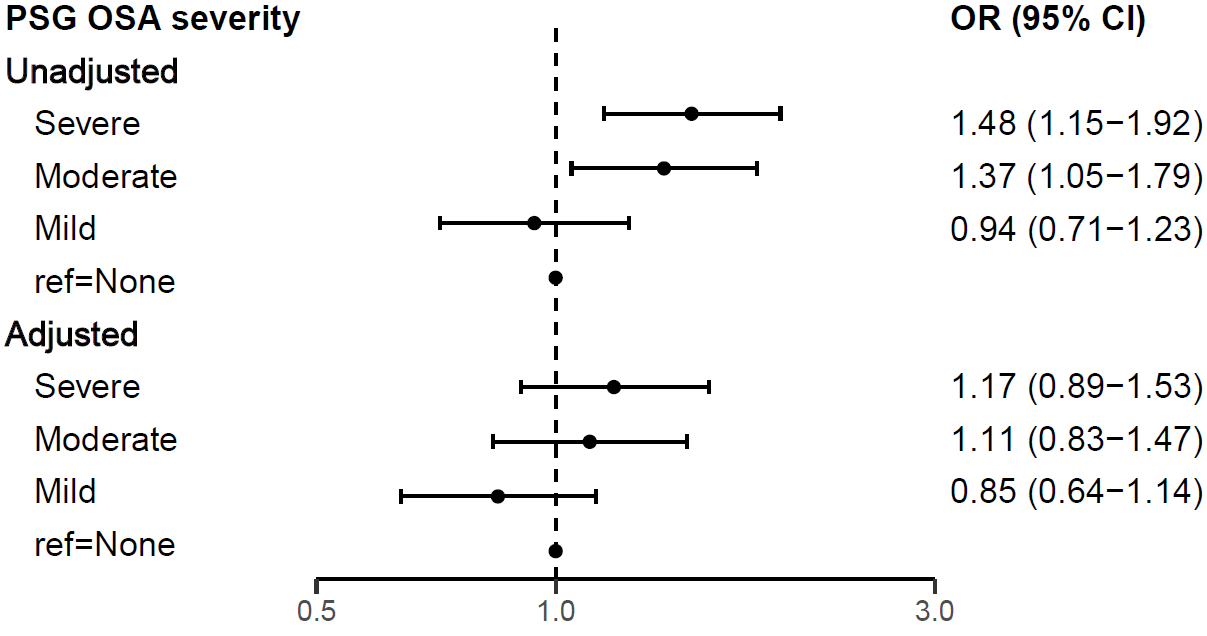


The Apnea-Hypopnea Index (AHI) was subsequently calculated as the average number of apneas and hypopneas per hour of sleep, classifying OSA severity as no OSA (AHI < 5), mild (AHI 5-14), moderate (AHI 15-29), and severe (AHI ≥ 30). The adjusted models account for BMI, age at the time of the PSG, race (white vs. non-white), sex, years of service, arrival time at the WTC site and smoking status (ever vs. never). High frequency HL was computed by averaging the lowest audible sound in the better ear across frequencies of 3 kHz, 4 kHz, and 6 kHz. Outcome measures included high-frequency hearing loss (≥35 dB)

**Supplemental table S4: Evaluating the association between Berlin Questionnaire OSA Risk and Speech Frequency Hearing Loss including confounders**

| **Parameter** | **OR (95% CI)** | **p-value** |
| --- | --- | --- |
| **Berlin risk** |  |  |
| 3 (very high) | 1.56 (1.30-1.88) | <0.01 |
| 2 (high) | 1.34 (1.14-1.58) | <0.01 |
| 1 (low) | 1.19 (1.01-1.41) | 0.04 |
| ref = 0 (none) |  |  |
| **Age at hearing exam** | 1.15 (1.14-1.16) | <0.01 |
| **Race** |  |  |
| White | 0.59 (0.48-0.71) | <0.01 |
| non-White | ref |  |
| **Years of service** | 0.98 (0.97-0.99) | <0.01 |
| **Smoking** |  |  |
| Current smoker | 1.38 (1.10-1.71) | <0.01 |
| Former smoker | 1.30 (1.16-1.46) | <0.01 |
| Never smoker | ref |  |
| **WTC arrival time** |  |  |
| Morning of 9/11/2001 | 0.95 (0.66-1.39) | 0.79 |
| Afternoon of 9/11/2001 | 0.95 (0.67-1.36) | 0.76 |
| 9/12/2001 | 0.90 (0.63-1.31) | 0.59 |
| 9/13/2001 | 0.86 (0.60-1.26) | 0.43 |
| After 9/24/2001 | ref |  |

**Legend**: Speech frequency HL was computed by averaging the lowest audible sound in the better ear across frequencies of 0.5 kHz, 1 kHz, 2 kHz, and 4 kHz. Outcome measures included hearing loss (≥20 decibels (dB)) at speech frequency averages.

**Note**: Models evaluating the OSA-HL association were adjusted for confounders; thus, all reported values reflect conditional associations.

**Supplemental table S5: Evaluating the association between OSA severity by PSG and Speech Frequency Hearing Loss including confounders**

| **Parameter** | **OR (95% CI)** | **p-value** |
| --- | --- | --- |
| **PSG OSA severity** |  |  |
| Severe | 1.33 (1.00-1.78) | 0.04 |
| Moderate | 1.13 (0.84-1.53) | 0.43 |
| Mild | 1.11 (0.83-1.49) | 0.47 |
| ref = None |  |  |
| **Age at hearing exam** | 1.07 (1.06-1.09) | <0.01 |
| **Race** |  |  |
| White | 0.62 (0.41-0.91) | 0.01 |
| non-White | ref |  |
| **Years of service** | 1.02 (1.00-1.03) | 0.01 |
| **Smoking** |  |  |
| Current smoker | 1.55 (1.27-1.89) | <0.01 |
| Former smoker | 1.19 (0.62-2.12) | 0.58 |
| Never smoker | ref |  |
| **WTC arrival time** |  |  |
| Morning of 9/11/2001 | 0.57 (0.27-1.26) | 0.16 |
| Afternoon of 9/11/2001 | 0.48 (0.24-1.06) | 0.07 |
| 9/12/2001 | 0.51 (0.24-1.12) | 0.09 |
| 9/13/2001 | 0.59 (0.28-1.32) | 0.19 |
| After 9/24/2001 | ref |  |
| **BMI** |  |  |
| Obese | 1.40 (0.90-2.25) | 0.14 |
| Overweight | 0.97 (0.62-1.58) | 0.91 |
| Normal | ref |  |

**Abbreviations**: OSA=Obstructive Sleep Apnea, PSG=Polysomnography, kHz=kilohertz

The Apnea-Hypopnea Index (AHI) was subsequently calculated as the average number of apneas and hypopneas per hour of sleep, classifying OSA severity as no OSA (AHI < 5), mild (AHI 5-14), moderate (AHI 15-29), and severe (AHI ≥ 30). Speech frequency HL was computed by averaging the lowest audible sound in the better ear across frequencies of 0.5 kHz, 1 kHz, 2 kHz, and 4 kHz. Outcome measures included hearing loss (≥20 decibels (dB)) at speech frequency averages.

**Note**: Models evaluating the OSA-HL association were adjusted for confounders; thus, all reported values reflect conditional associations.
